# Notes on the genus *Theopropus* Saussure (Mantodea, Hymenopodidae) from China, with description of a new species from the Himalayas

**DOI:** 10.3897/zookeys.1049.65295

**Published:** 2021-07-23

**Authors:** Chao Wu, Chun-Xiang Liu

**Affiliations:** 1 Key Laboratory of the Zoological Systematics and Evolution, Institute of Zoology, Chinese Academy of Sciences, Beichen West Road, Chaoyang District, Beijing 100101, China Institute of Zoology, Chinese Academy of Sciences Beijing China

**Keywords:** Distribution, mantis, new subspecies, Oriental region, taxonomy

## Abstract

The genus *Theopropus* Saussure, 1898 is distributed with five species in SE Asia, three of which were recorded in South China: *T. elegans* (Westwood), *T. cattulus* (Westwood) and *T. sinecus* Yang. After examining numerous specimens of *Theopropus*, we attempt to resolve some taxonomic confusion about *Theopropus* occurring in China. Those Chinese specimens that were inaccurately identified as *T. cattulus* represent a new subspecies of *T. sinecus* Yang: *T. s. qiongae* Wu & Liu, **ssp. nov.**. A new species, *T. xishiae* Wu & Liu, **sp. nov.**, is described from the rainforests of the southern slopes of the Himalayas. The records of *T. elegans* in China are also clarified. Biological characteristics of the species and subspecies, necessary illustrations, and ecological images are provided. The distribution of the known Chinese *Theopropus* species is discussed and mapped.

## Introduction

The genus *Theopropus* Saussure, 1898 was erected for *Blepharis elegans* Westwood, 1832, the type of which was collected in Tanesserim, Myanmar. Previously it included five species (Yang 1999; Otte and Spearman. 2005): *T. borneensis* Beier, 1942 recorded from Borneo, *T. cattulus* (Westwood, 1889) described from Java, *T. sinecus* Yang, 1999 described from South China, *T. elegans* (Westwood, 1832) widely distributed in Southeast Asia, and *T. rubrobrunneus* Beier, 1931 described from Malaysia. Additionally, two taxa were also listed as synonyms of *T. elegans* (Otte & Spearman, 2005): T. elegans var. flavicans Giglio-Tos, 1927 and *T. praecontatrix* Saussure, 1898, although T. elegans var. rubrobrunneus Beier, 1931 was also considered as synonym of *T. elegans* in the research of Beier (1934) and of Ehrmann (2002).

*Theopropus* is widely distributed in southern China, the Indochinese Peninsula, and the Malay Archipelago. In China, *T. elegans* was first mentioned to be distributed in Yunnan by Tinkham (1937), but without specimen records. The report of *T. elegans* from Yunnan in Tinkham (1937) was questioned by Wang (1993) and Yang and Wang (1999) because no specimens were examined. Afterwards, a male specimen of *T. elegans* was reported from Wuyishan Mountain in Fujian Province by Wang (1993). Subsequently, the new species *T. sinecus* was described by Yang (1999) based on a female (holotype) and a male (paratype) specimen, which were collected from Jinxiu in Guangxi Province. Additionally, *T. cattulus* Westwood was reported to be distributed in Hainan Island by Zhu et al. (2012), who also noted that *T. sinecus* was a synonym of *T. elegans*, but without standard taxonomic treatment. As a consequence, there is considerable taxonomic confusion concerning the common and attractive mantis genus *Theopropus* in China.

In this research, we examined numerous specimens, which were collected in China and neighboring countries, aiming to illustrate the taxonomic situation of the genus *Theopropus* in China. We clarified the validity of *Theopropus sinecus* Yang, the distributions of *T. elegans* and *T. cattulus*, redescribed the known Chinese species and describe a new taxon.

## Materials and methods

Classification system follows Schwarz and Roy (2019). Descriptive terminology of adult morphology and the male genitalia follows Brannoch et al. (2017) and Schwarz and Roy (2019). Specimens were collected during the daytime through careful observation or by light trap (male). Genitalia were dissected in 10% KOH solution, cleared with pure water, and finally stored in 70% ethanol in Eppendorf tubes for further research. Pictures were taken with a Nikon digital camera.

The specimens were deposited in the following institutions or private collections.

**CAU**China Agricultural University, Beijing, China;

**CJZ** Collection of Jia-Zhi Zhang, Shanghai, China;

**CWC** Collection of Chao Wu, Beijing, China;

**IZCAS**Institute of Zoology, Chinese Academy of Sciences, Beijing, China.

## Taxonomic treatment

### Order Mantodea Wood-Mason, 1889

#### Family Hymenopodidae Giglio-Tos, 1915

**Subfamily Hymenopodinae Giglio-Tos, 1915**

**Tribe Hymenopodini Giglio-Tos, 1915**

##### Genus Theopropus

Taxon classificationAnimaliaMantodeaHymenopodidae

Saussure, 1898

CB03A961-1912-51B7-BC50-42F4177E6650

Figs 1 , 2 , 3 , 4 , 5 , 6 , 7 , 8 , 9 , 10 , 11 , 12 , 13 , 14

Theopropus Saussure, 1898: 204; Kirby 1904: 293; Giglio-Tos 1915: 106; Giglio-Tos 1927: 561; Beier 1934: 27; Beier 1942: 152; Beier 1964: 939; Beier 1968: 6; Ehrmann 2002: 353; Otte and Spearman 2005: 99; Zhu et al. 2012: 52; Schwarz and Konopik 2014: 145; Schwarz and Roy 2019: 118, 152.

###### Type species.

*Blepharis elegans* Westwood, 1832

**Figure 1.**
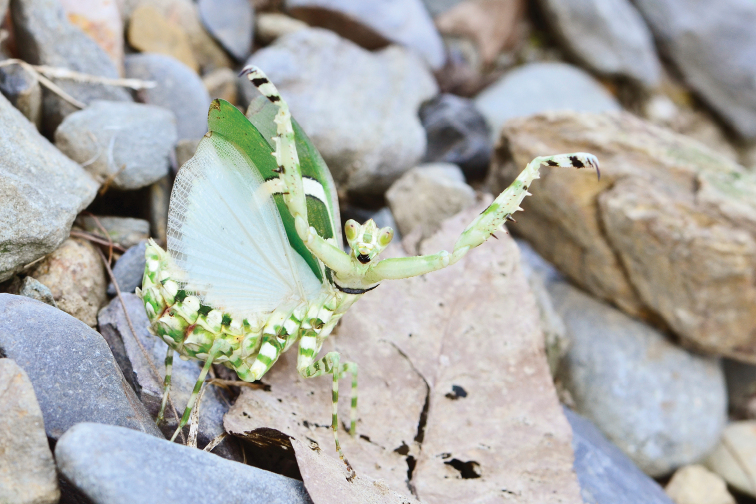
Female *Theopropus sinecus sinecus* in natural habitat, from Guangxi.

**Figure 2.**
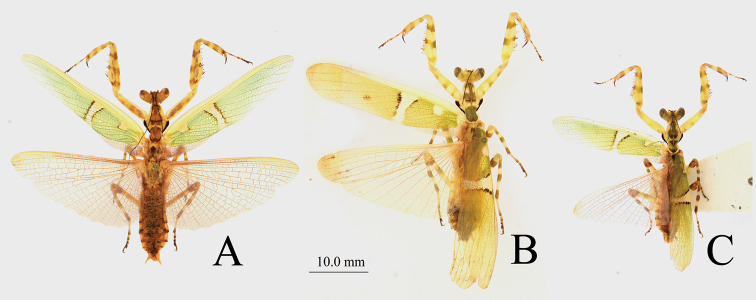
Male *Theopropus* spp. body in dorsal view **A***T. sinecus sinecus* from Guangxi **B***T. xishiae* sp. nov. paratype **C***T.* sp. from Yunnan.

**Figure 3.**
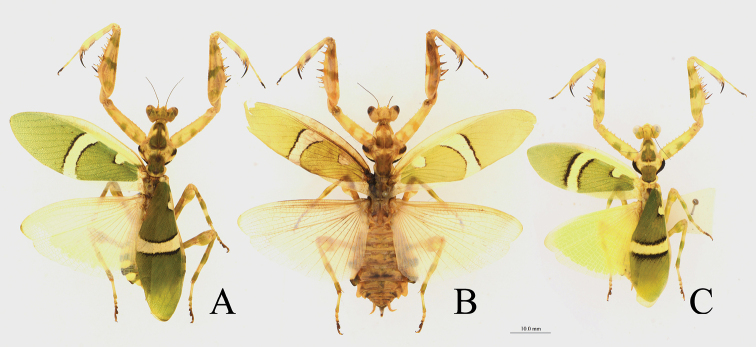
Female *Theopropus* spp. body in dorsal view **A***T. sinecus sinecus* from Guangxi **B***T. xishiae* sp. nov. paratype **C***T.* sp. from Yunnan.

**Figure 4.**
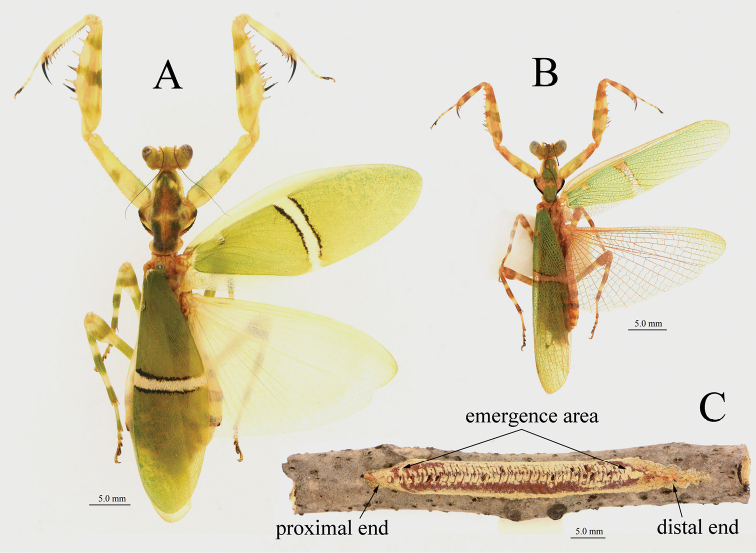
*Theopropus sinecus qiongae* ssp. nov. body in dorsal view and ootheca **A** female, paratype **B** male, paratype **C** ootheca.

###### Diagnosis.

Medium to large-sized Hymenopodidae, with mottled body coloration. Male and female distinctly differing by body size, male body smaller, often shorter than half body length of females.

**Figure 5.**
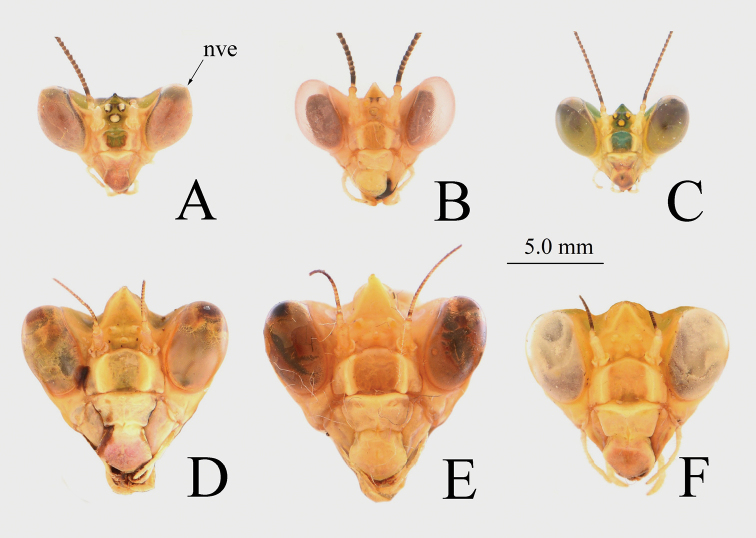
Head of *Theopropus* spp., anterior view **A, D***T. sinecus sinecus***B, E***T. xishiae* sp. nov. **C, F***T.* sp. **A–C** male **D–F** female. Abbreviations: **nve** = non-visual elongation.

***Head*** (Fig. 5): Triangular. Compound eyes oval, convex, uprising beyond vertex. Vertex with a robust vertical process, coniform. Lower frons wider than high. Antennae filiform, shorter than body length; antennae thick and long in males, thin and short in females.

**Figure 6.**
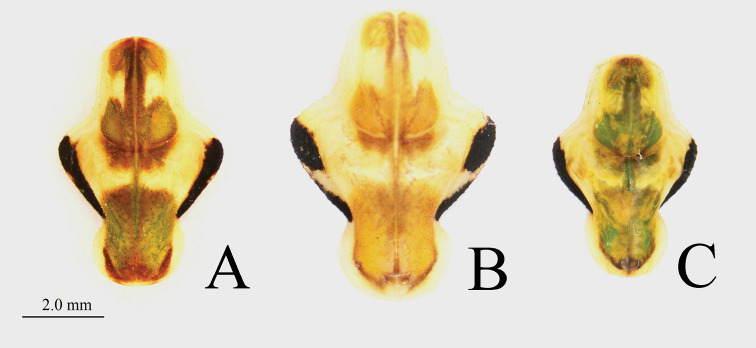
Pronotum of male *Theopropus* spp. in dorsal view **A***T. sinecus sinecus* from Guangxi **B***T. xishiae* sp. nov. holotype **C***T.* sp. from Yunnan.

**Figure 7.**
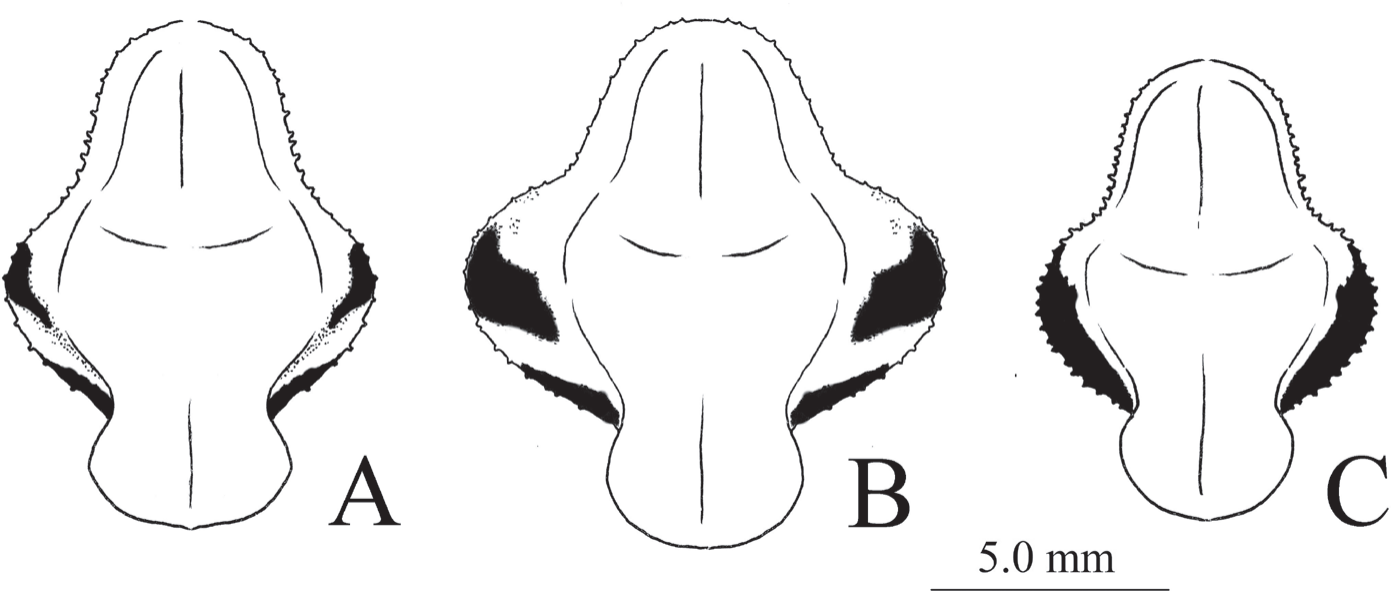
Pronotum of female *Theopropus* spp. in dorsal view **A***T. sinecus sinecus* from Guangxi **B***T. xishiae* sp. nov. paratype **C***T.* sp. from Yunnan.

***Pronotum*** (Figs 6, 7): Short, wide, with obvious lateral pronotal expansion at transverse groove, prozone slightly shorter than metazone. Lateral margins of pronotum with small spines.

**Figure 8.**
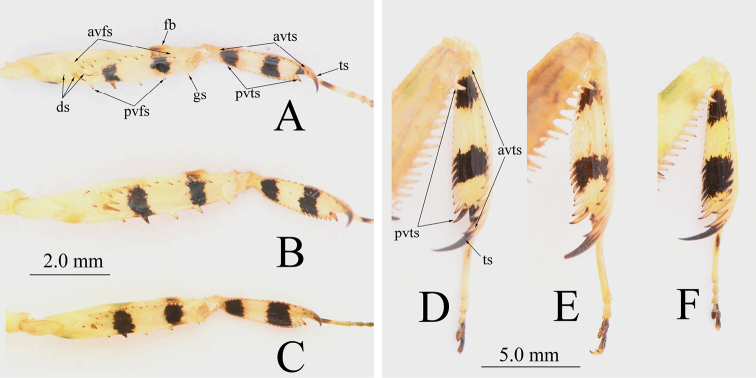
Prothoracic legs of *Theopropus* spp. **A, D***T. sinecus sinecus***B, E***T. xishiae* sp. nov. **C, F***T.* sp. **A–C** male **D–F** female. Abbreviations: **avfs** = anteroventral femoral spines; **avts** = anteroventral tibial spines; **ds** = discoidal spines; **fb** = femoral brush; **gs** = genicular spur; **pvfs** = posteroventral femoral spines; **pvts** = posteroventral tibial spines; **ts** = tibial spur.

***Prothoracic legs*** (Fig. 8): Long, robust; coxa distinctly longer than pronotum, with small dorsal spines. Femora with 4 posteroventral, 3 discoidal and about 15–20 anteroventral spines; tibia with about 15–20 anteroventral and posteroventral spines, posteroventral spines decumbent.

**Figure 9.**
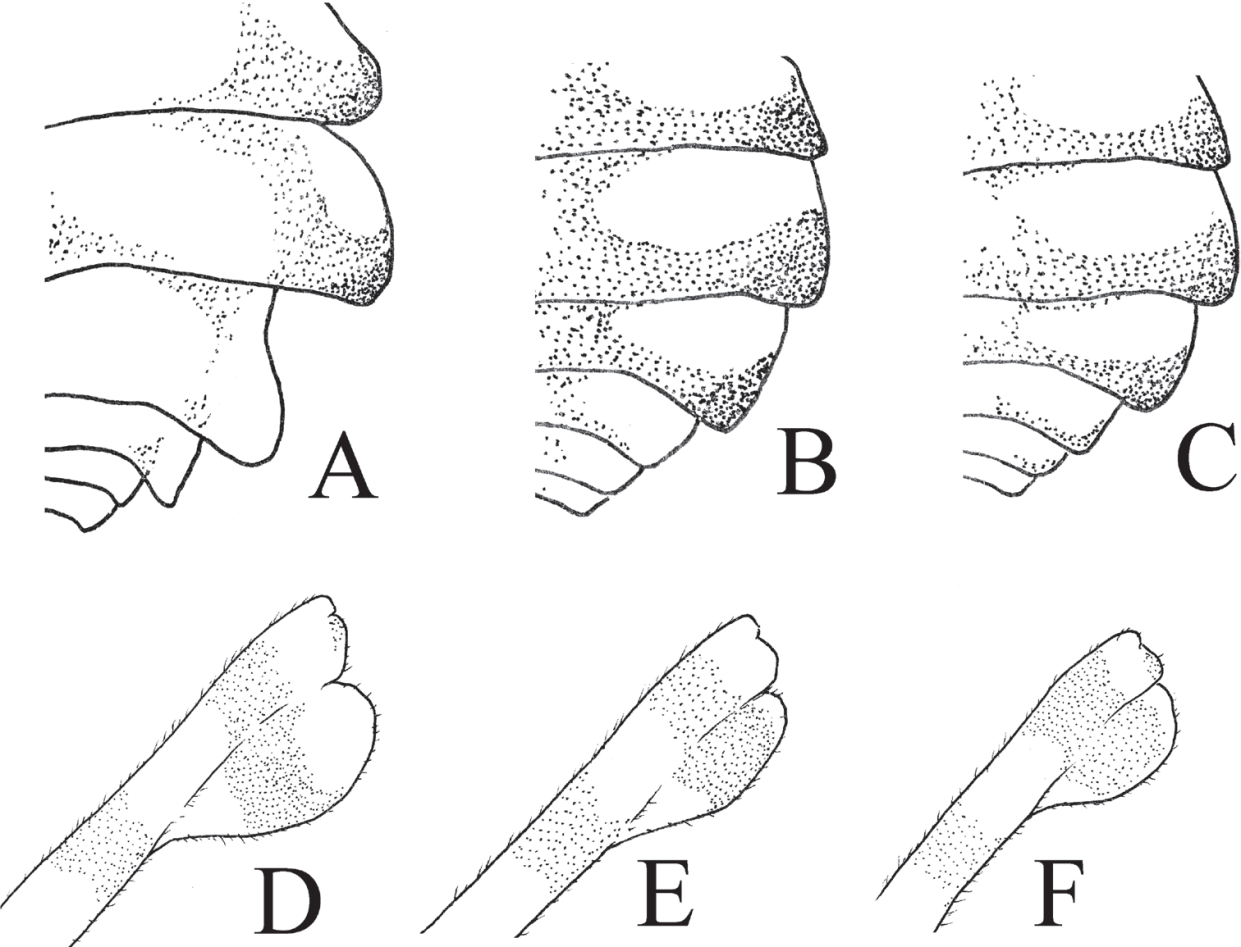
Abdomen and posteroventral metafemoral lobe of female *Theopropus* spp. **A, D***T. xishiae* sp. nov. **B, E***T. sinecus sinecus***C, F***T.* sp. **A–C** abdomen **D–F** posteroventral metafemoral lobe.

***Meso- and metathoracic legs*:** Long, robust; subapical part of the femur with a posteroventral lobe (Fig. 9D–F). Base half of tibia swollen.

***Wings*:** Forewings opaque, narrow, long in males, wide, fusiform in females; a white spot lying subbasally in the discoidal area; a white band with black borders on both lateral margins lying in middle of the discoidal area; anal area long, narrow. Hindwings broad, shorter than forewings; transparent or with opaque areas in males, subopaque in females.

***Abdomen*:** Narrow, long in male, wide in female. Cerci short, hairy. Male subgenital plate short, wide, with small styli.

**Figure 10.**
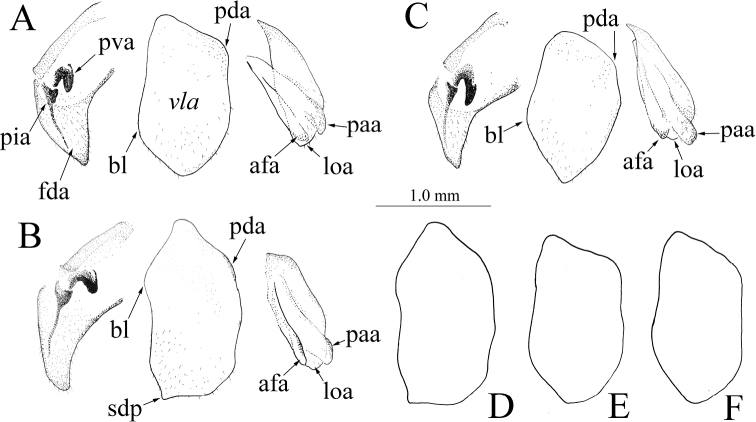
Male genitalia of *Theopropus* spp., Disarticulated genital complex, ventral view **A***T. sinecus sinecus* from Guangxi, Jinxiu **B***T. xishiae* sp. nov. holotype **C***T.* sp. from Yunnan, Mengla **D***T. xishiae***sp. nov.** paratype **E***T. sinecus sinecus* from Yunnan, Honghe **F***T. sinecus qiongae* ssp.nov. holotype. Abbreviations: **afa** = phalloid apophysis; **bl** = basal lobe of ventral phallomere; **fda** = main posterior lobe of right phallomere; **loa** = membra- nous lobe; **paa** = posterior process of left phallomere; **pda** = primary distal process; **pia** = process posterolateral to pva of right phallomere; **pva** = process anteromesal to pia of right phallomere; **sdp** = secondary distal process.

***External genitalia*** (Fig. 10A–F): Male genitalia simple, similar among congeners. Secondary distal process reduced.

**Figure 11.**
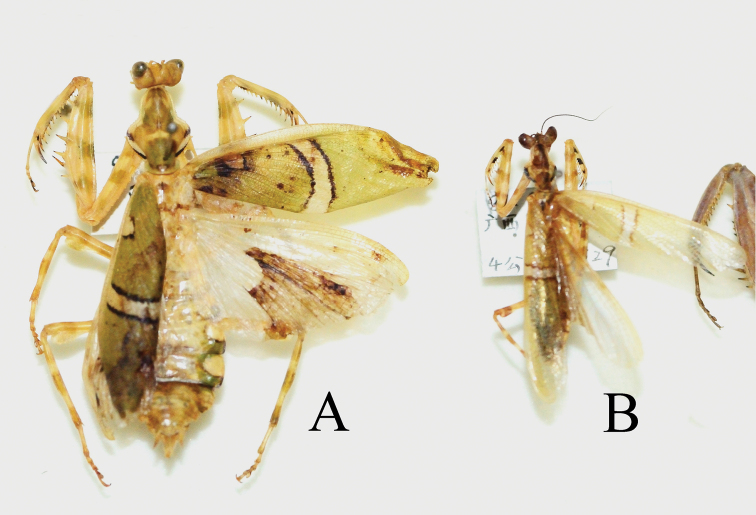
Holotype and paratype of *Theopropus sinecus* Yang, 1999 **A** holotype, female **B** paratype, male.

***Ootheca*** (Fig. 4C): Very elongated, flat, narrowing at both ends.

###### Discussion.

The characteristics for the head, pronotum, and the range of the ratio of the pronotum length to supracoxal dilatation width are relatively stable in the species; these characteristics can be used to identify species. The male genitalia of *Theopropus* lack sclerotized projections and show little differences between species.

###### Distribution.

**Figure 12.**
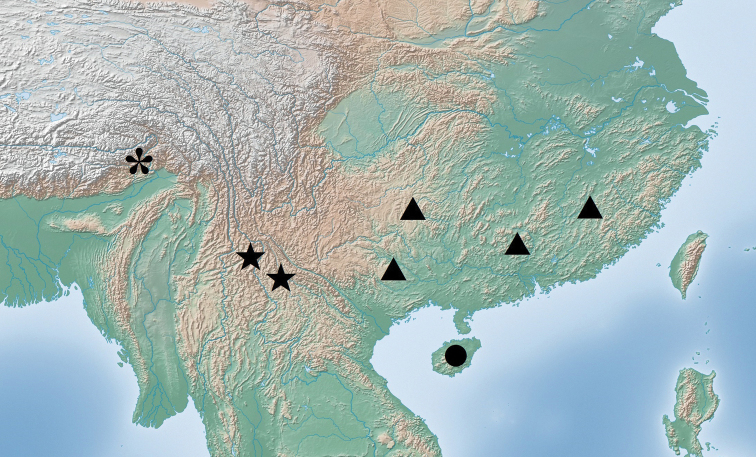
Distribution map of the distribution *Theopropus* spp. in South China. ▲: *T. sinecus sinecus*; ●: *T. sinecus qiongae* ssp. nov.; * *T. xishiae* sp. nov.; ★: *T.* sp..

The genus *Theopropus* is distributed in the tropical areas of southern Asia. In China, *Theopropus* species are widely distributed in South and Southwest China (Fig. 12).

##### Theopropus sinecus

Taxon classificationAnimaliaMantodeaHymenopodidae

Yang, 1999

B8F35A7E-4F20-5B33-BC26-F6011D0EA835

Theopropus sinecus Yang, 1999: 28; T. elegansZhu et al. 2012: 52–55.

###### Comments.

*Theopropus sinecus* Yang is widely distributed in South China. Its types were collected from Guangxi Province. Specimens from the mainland and Hainan Island have similar body features and were mated to produce fertile offspring in our own breeding facilities, but they clearly differ by body color and spots’ characteristics in the forewings. Therefore, we consider specimens from Hainan Island as a new subspecies for *T. sinecus* Yang.

##### Theopropus sinecus sinecus

Taxon classificationAnimaliaMantodeaHymenopodidae

Yang, 1999 sensu str.

B91408AB-8B3D-5100-9600-06C992323031

Figs 1 ; 2A ; 3A ; 5A, D ; 6A ; 7A ; 8A, D ; 9B, E ; 10A, E ; 11 ; 12 ; 13A, B ; 14C

###### Type locality.

China: Guangxi, Jinxiu.

###### Material examined.

17♂, 15♀. ***Holotype*. China** • 1♀, Guangxi, Jinxiu; 18-XI-1981, No type label, CUA; • 1♂ ***Paratype*.** Guangxi, Jinxiu; 21-IX-1981, No type label, CUA; • 1♂; Guangxi, Longsheng, Huaping; 980 m; IX-2009; Ye Liu leg.; IZCAS; • 3♂; Guangxi, Longsheng, Huaping; 25°37'28"N, 109°54'07"E; 900–1000 m; 15~20-IX-2012; Chao Wu leg.; CWC; • 5♀; same as before; • 2♂; Guangxi, Guilin; 25°19'04"N, 110°23'24"E; 700 m; 13-X-2014; Chao Wu leg.; CWC; • 1♀; Guangxi, Jinxiu, Lianhuashan Mt.; 950 m; 30-IX-2014; Chao Wu leg.; IZCAS; • 2♂; same as before; • 1♂; Guangdong, Shaoguan, Nanling Mt.; 24°56'30"N, 113°01'07"E; 1000 m; 10-X-2011; IZCAS; • 1♀; Fujian, Nanping, Wuyishan Mt.; 27°42'25"N, 117°39'04"E; 1200 m; 15-VII-2020; Zhao-Nan Xia leg.; CJZ; • 2♀, 5♂; Fujian, Nanping, Wuyishan Mt.; 27°43'N, 117°40'E; 800–1000 m; 13-VIII-2019; Zhao-Nan Xia leg.; CWC; • 1♀, 1♂; Fujian, Nanping, Wuyishan Mt.; 27°43'N, 117°40'E; 800–1000 m; 9-IX-2020; Chao Wu leg.; CWC; • 1♀; Hubei, En’shi, Lichuan, Liangwu; 1300 m; 8-VIII-2018; Chuan Qin leg.; CJZ; • 1♂; Yunnan, Honghe, Hekou; 22°39'12"N, 103°58'52"E; 800 m; 15-XI-2017; Chao Wu leg. CWC; • 2♀ same as before. **Vietnam** • 1♀; N-Vietnam; VIII-2001; other information unknown; CWC.

###### Redescription.

**Male.** Large-sized compared with congeners, body length reaching half that of females.

***Head*:** Compound eyes oval, anteriorly protruding, with a very weak conical elongation at tip (Fig. 5A). Vertical process distinct, coniform, lower than the apex of compound eyes (Fig. 5A). Lower frons with arched superior margins and raised lateral margins. Antennae filiform, shorter than body length.

***Pronotum*:** Short, lateral pronotal expansion wide. Ratio of pronotum length to supracoxal dilatation width about 1.49–1.53. Lateral margins of the pronotum indistinctly granulated. Lateral margin of metazone with continuous black edge (Fig. 6A).

***Prothoracic legs*:** Coxa bearing 6–9 small dorsal spines, femora bearing 15–16 anteroventral spines, tibia bearing 15–16 anteroventral and 18–19 posteroventral spines.

***Meso- and metathoracic legs*** (Figs 2A, 3A): posteroventral genicular lobe on femur long, narrow (Fig. 9E). Base half of tibia swollen.

***Wings*** (Figs 2A, 3A): Forewings narrow, long. A wide white band lying in middle of discoidal area; two black parallel arc-shaped borders at lateral margins of the wide white band directing proximad, “))”-shaped; anal area narrow, long, transparent. Hindwings broad, hyaline.

***Abdomen*:** Long, narrow, without lobe. Subgenital plate short, wide, more or less asymmetrical, with styli.

***External genitalia*:** Simplified. Left phallomere wide, secondary distal process (spd) indistinct; phalloid apophysis (afa) short; posterior process of left phallomere (paa) digitiform (Fig. 10A).

**Female.** Similar to male, but body more robust, size larger than that of male. Vertical process distinct, conical, extending beyond apex of eyes (Fig. 5D). Pronotum wide, lateral pronotal expansion very wide, ratio of pronotum length to supracoxal dilatation width about 1.37–1.40; black band on each lateral margin of metazone traversed by a white band (Fig. 7A). Forewings wide, fusiform, extending beyond abdominal apex. Hindwings broad, opaque. Abodomen broad, nearly rounded, 4^th^-6^th^ abdominal tergite inconspicuously slightly expanded laterad.

###### Coloration.

Overall green, with white spots and bands. Antennae black. Lateral margin of metazone band black. The two horizontal ventral bands on prothoracic femora and tibia black in male (Fig. 8A), absent on femora for females (Fig. 8D). A black ventral spots near prothoracic tibial spur (Figs 6A, 7A). Forewings green, costal area white, discoidal area green; the large subbasal spot of discoidal area white, with black hind edge; wide band in middle of discoidal area white, with black lateral margins; anal area orange in males and white in females. Hindwings orange with red-brownish veins in males, whitish to slightly yellowish with hyaline margin in females. Abdomen yellowish white, plaques on lateral margins of 2^th^-6^th^ abdominal tergite green (Fig. 9B).

###### Measurements

**(length in mm).** Body (head to wings): male 28.2–29.3, female 46.3–47.3; body (vertex to abdomen end): male 25.6–27.3, female 44.1–47.1; pronotum: male 5.9–6.0, female 11.9–12.1; fore coxae: male 5.9–6.1 , female 13.0–13.7; fore femora: male 7.2–7.3, female 15.0–15.5; fore tibiae: male 5.4–5.41, female 11.1–11.4; middle femora: male 6.0–6.1 , female 10.8–11.0; hind femora: male 7.1–7.2, female 13.0–13.5; forewing: male 21.1–21.8, female 29.9–31.8; hindwing: male 18.9–19.2, female 25.0–26.0.

###### Note.

When examining numerous specimens from Wuyishan, Fujian Province (listed above), we found that they are the same as the types of *Theopropus sinecus*. Their body is larger than in *T. elegans* from Malaysia; the compound eyes possess a conical elongation at the tip in males; the black band on each lateral margin of the pronotum is continuous in males, but disconnected in females. In *T. elegans*, the compound eyes do not have a conical elongation in males, and the black band on the lateral margin of the pronotum is contiguous in both sexes. Also, female hindwings are orange with smoky margins in *T. elegans*. The specimens of *Theopropus* from Wuyishan should therefore be identified as *T. sinecus sinecus*. We think that the record of *T. elegans* (in Wuyishan, Fujian Province) might have been mistaken by Wang (1993) and Wang and Yang (1999).

Yang (1999) wrote the specimen information in the Chinese description as “Guangxi-Dayaoshan Mt., 1981-VIII, Qijing You leg.”, however, the specimens of *Theopropus* with the same collection information could not be found among Yang’s research specimens. Only one female labeled “Guangxi, Jinxiu; 18-XI-1981” and one male labeled “Guangxi, Jinxiu; 21-IX-1981” could be found. We re-measured these specimens and obtained the following measurements: length of body (head to wings) about 29.3 in male and 45.2 in female, length of forewings about 21.6 in male and 29.2 in female (impaired), in original description, length of body (head to wings) 31 in male and 44 in female, length of forewings about 22 in male and 29 in female. In addition, as the illustration (hand-painted) of the original description is similar to the posture of the female specimen, we confirm that these two specimens are the types of *T. sinecus* Yang.

###### Distribution.

China: Guangxi, Guangdong, Yunnan, Fujian, Hubei; Vietnam.

##### Theopropus sinecus qiongae

Taxon classificationAnimaliaMantodeaHymenopodidae

Wu & Liu ssp. nov.

00F2783E-40DE-5218-8FCD-C5A097FF5FAE

**Figure 13.**
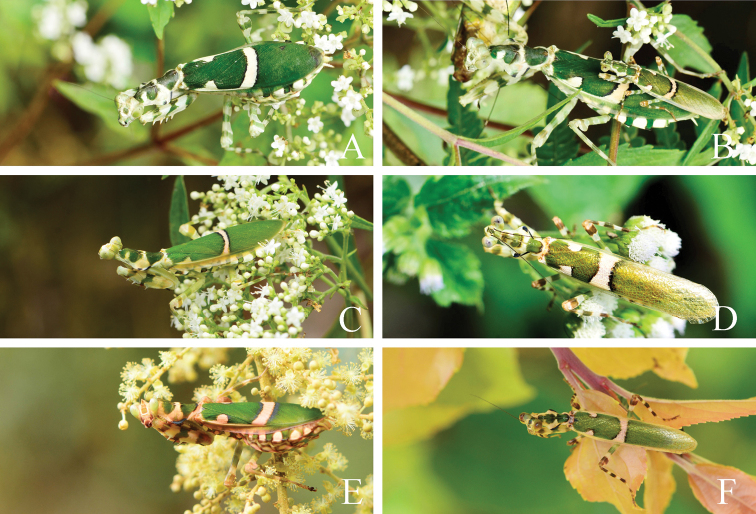
*Theopropus* spp. adult in its natural habitat **A, B***T. sinecus sinecus***C***T. sinecus qiongae* ssp. nov. **D***T. xishiae* sp. nov. **E, F***T.* sp. **A, C, E** female **B** pair **D, E** male.

**Figure 14.**
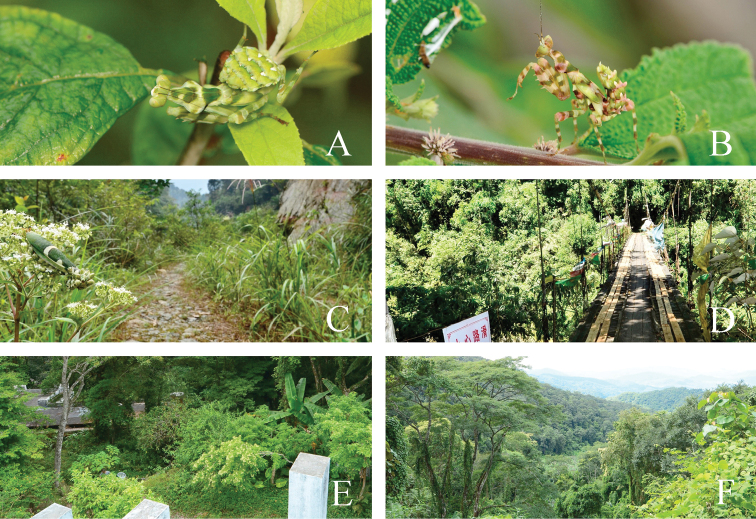
*Theopropus* spp. nymph in its natural habitat and environment **A***T. sinecus qiongae* ssp. nov. female nymph in Hainan Is **B***T. xishiae* sp. nov. male nymph in Medog **C***T. sinecus sinecus* of Guangxi, Longsheng, Huaping **D***T. xishiae* sp. nov. of Tibet, Medog, Beibeng **E***T. sinecus qiongae* ssp. nov. of Hainan Is. Jianfengling Mt. **F***T.* sp. of Yunnan, Mengla, Mohan.

Figs 4 ; 10F ; 12 ; 13C ; 14A, E

Theopropus cattulus , Zhu et al. 2012: 56–58 (erroneously identified).

###### Material examined.

10♂, 6♀. ***Holotype.* China** • 1♂; Hainan, Ledong, Jianfengling Mt., Mingfenggu; 18°44'75"N, 108°50'28"E; 950 m; 30-VI-2020; Chao Wu leg.; IZCAS. ***Paratypes.*** China • 2♀; Hainan, Ledong, Jianfengling Mt., Tianchi; 18°44'25"N, 108°51'37"E; 900 m; 15-XI-2016; Chao Wu leg. IZCAS. • 1♀; Hainan, Ledong, Jianfengling Mt., Tianchi; 10-IV-2010; Xin-Lei Huang leg. IZCAS. • 1♀; Hainan, Baisha, Shuiman, Wuzhishan Mt.; 18°53'17"N, 109°40'01"E; 750 m; 20-VII-2020; Chao Wu & Cai-Wen Nie leg.; CWC. • 1♀; Hainan, Baisha, Hongkan, Ying’geling Mt.; 600 m; 23-X-2014; Chao Wu leg.; CWC. • 3♂; same as before; IZCAS. • 4♂; Hainan, Qiongzhong, Limushan Mt.; 700 m; 20-X-2014; Chao Wu leg.; CWC. • 1♂ Hainan, Ledong, Jianfengling Mt., Mingfenggu; 950 m; 28-X-2014; Chao Wu leg.; CWC. • 1♀; Hainan, Ledong, Jianfengling Mt., Mingfenggu; 20-XII-2017; Jia-Zhi Zhang leg.; CJZ. • 1♂; same as before.

###### Description.

**Male.** Similar to *T. s. sinecus* (Figs 4A, B, 10F), except the following characteristics: smaller; on surface of forewing, the white subbasal spot of discoidal area small, even disappearing in some specimens; white band at the middle of discoidal area narrow, the two black parallel arc-shaped lateral borders of the white band converging at their distal ends in rare instances; hindwing base reddish brown, with red-brownish veins.

**Female.** Similar to *T. s. sinecus* but smaller. The forewing is similar to male, white spot in base of discoidal area small or indistinct, white band in middle of discoidal area narrow; hindwings opaque, maize-yellow, with hyaline margin.

###### Discussion.

Zhu et al. (2012) identified the specimens from Hainan Island as *Theopropus cattulus* (Westwood, 1889). After checking the pictures of the types of *T. cattulus* Westwood, 1889 in “The Mantodea Image Database” https://specimens.mantodearesearch.com/default/zoom/835, we found that there is only a small black spot on each lateral margin of the metazone in *T. cattulus*, whereas, in those specimens from Hainan Island, a black band extends backwards on each lateral margin of metazone. Also, *Theopropus cattulus* is endemic on Java, Indonesia. Thus, we think the identification of these specimens from Hainan Island in Zhu et al. (2012) is wrong. We establish a new subspecies of *T. sinecus* for these specimens from Hainan Island.

###### Measurements

**(length in mm, holotype in parentheses).** Body (head to wings): male 27.9–28.6 (28.5), female 44.9–46.2; body (vertex to abdomen end): male 22.0–26.5 (25.8), female 40.3–42.1; pronotum: male 5.8–6.0(5.9), female 11.6–11.9; fore coxae: male 5.8–6.0 (5.9), female 12.88–13.02; fore femora: male 6.9–7.1 (7.0), female 14.0–14.9; fore tibiae: male 5.2–5.3 (5.2), female 10.4–10.9; middle femora: male 5.9–6.0 (5.9), female 10.3–10.6; hind femora: male 7.0–7.2 (7.1), female 12.0–13.1; forewing: male 20.0–20.1 (20.1), female 28.5–30.0; hindwing: male 18.2–18.9 (18.6), female 24.9–25.9.

###### Distribution.

China: Hainan Island.

###### Etymology.

The new subspecies was named after the other name for Hainan Island, Qiong.

##### Theopropus xishiae

Taxon classificationAnimaliaMantodeaHymenopodidae

Wu & Liu sp. nov.

70AF27FE-DB08-517B-9C19-F7C17C3D781D

http://zoobank.org/15E6FA92-601D-402F-A605-0B822A1CE170

Figs 2B ; 3B ; 5B, E ; 6B ; 7B ; 8B, E ; 9A, D ; 10B, D ; 12 ; 13D ; 14B, D

###### Material examined.

6♂, 1♀, 1♀ juv.**. *Holotype.*** China • 1♂; Tibet, Medog, Beibeng; 29°14'58.14"N, 95°10'31.55"E; 960 m; 12-VII-2013; Chao Wu leg.; IZCAS. ***Paratypes.*** China • 1♀; Tibet, Medog, Beibeng; 29°14'58.14"N, 95°10'31.55"E; 960 m; 12-VII-2013; Chao Wu leg.; IZCAS. • 2♂; Tibet, Medog, Dexing cun; 29°19'36.48"N, 95°16'59.82"E; 770 m; 15-VII-2013; Chao Wu leg.; IZCAS. • 2♂; Tibet, Medog, Jiangxin cun; 29°13'02.90"N, 95°08'05.61"E; 1200 m; 20-VII-2014; Chao Wu leg.; CWC. • 1♂; Tibet, Medog, Beibeng; 29°14'21.52"N, 95°12'00.21"E; 1320 m; 24-VII-2019; Chao Wu leg.; CJZ. • 1♀ juv.; Tibet, Medog, Beibeng; 1000 m; VII-2010; Wen-Xuan Bi leg.; IZCAS.

###### Description.

**Male.** Large-sized species for *Theopropus*. Body size much larger than in other congeners.

***Head*:** Compound eyes oval, anteriorly protruding, with rounded top. Vertical process conical, extending about as high as the imaginary line between the apexes of the eyes; lower frons narrow, with arched dorsal margin and raised lateral margins (Fig. 5B). Antennae filiform, shorter than body length.

***Pronotum*** (Fig. 6B): Wide. Lateral pronotal expansion very wide; lateral margins bearing small, sparsely arranged spines. Black band on each lateral margin of metazone disconnected in middle. Ratio of pronotum length to supracoxal dilatation width about 1.39–14.2.

***Prothoracic legs*** (Fig. 8B): Coxa bearing 6–7 small dorsal spines, femora with 15 anteroventral spines, tibia with 15 anteroventral and 18 posteroventral spines.

***Meso- and metathoracic legs*:** Long, robust; a subapical posteroventral lobe on mid and hind femora, narrow, long. Base half of tibia swollen.

***Forewings*:** Long, narrow, opaque. Discoidal area possessing a large subbasal white spot with blurry black edges; the two black lateral borders of the wide white band in middle of the discoidal area arched, the anterior margin of the frontal one directing proximad, of the caudal one directing distad (Fig. 13D). Anal area long, narrow, hyaline.

***Hindwings*** (Fig. 2B): Hyaline.

***Abdomen*:** Long, narrow, with very small lobes. Subgenital plate short, wide, more or less asymmetrical, with styli.

***External genitalia*** (Fig. 10B, D): Simplified. Similar to those of congeners. Left phallomere wide with inconspicuous secondary distal process (spd); phalloid apophysis (afa) short; posterior process of left phallomere (paa) digitiform.

**Female.** Large-sized, robust. Body size largest among known *Theopropus* species.

***Head*** (Fig. 5E): Similar to male, but vertex extending beyond apex of eyes.

***Pronotum*** (Fig. 7B): Wide. Lateral pronotal expansion very wide. Lateral margins bearing small, sparsely arranged spines. Black band on each lateral margin of metazone disconnected in middle. Ratio of pronotum length to supracoxal dilatation width about 1.08.

***Prothoracic legs*** (Fig. 8E): Coxa bearing 7–8 small dorsal spines; femora with 16 anteroventral spines; tibia with 16 anteroventral and 19 posteroventral spines. Two black horizontal bands present on ventral side of tibia, but absent in femora. No black spots observed near tibial spur.

***Meso- and metathoracic legs*:** Long, robust. The subbasal posteroventral lobe on femur wide, disc-shaped (Fig. 9D); base half of tibia swollen.

***Forewings*** (Fig. 3B): Wide, fusiform, opaque. The large white subbasal spot of the discoidal area with black edges; frontal one of the two black lateral margins of the wide white band in the middle of discoidal area arc-shaped, pointing proximad, and hind one approximately straight. Anal area long, narrow, hyaline.

***Hindwings*** (Fig. 3B): Wide, opaque, except for margin.

***Abdomen*** (Fig. 9A): Broad, nearly round. Lateral margins of 4^th^-7^th^ abdominal tergite with significantly expanded lobes.

###### Coloration.

Yellowish green, with white spots and bands. Antennae black. Band on each lateral margin of metazone black. Two horizontal ventral bands on prothoracic femora and tibia black in males (Fig. 8B), which is absent on femora in females (Fig. 8E). Forewings yellowish green, costal area white; the large spot in base of discoidal area white, with black hind edge; the wide band in middle of discoidal area white, with black lateral margins; anal area orange in males and white in females. Hindwings hyaline, with red-brownish veins in males, ivory in females. Abdomen yellowish white; lateral margins of 3^th^-5^th^ abdomen tergite with green plaques, and 6^th^-7^th^ mostly white in females.

###### Measurements

**(length in mm, holotype in parentheses).** Body (head to wings): male 33.0–33.7 (33.7), female 52.45; body (vertex to abdomen end): male 27.2–28.1, female 49.1; pronotum: male 6.3–6.4 (6.4), female 13.4; fore coxae: male 6.6–6.7 (6.7), female 13.6; fore femora: male 7.4–7.5 (7.5), female15.5; fore tibiae: male 5.3–5.4 (5.4), female 11.4; middle femora: male 6.8–6.9 (6.9), female 11.1; hind femora: male 8.0–8.1 (8.1), female 13.6; forewing: male 25.0–25.2 (25.2), female 35.1; hindwing: male 22.0–22.2 (22.2), female 29.8.

###### Differential diagnosis.

The new species most resembles *Theopropus sinecus*. It is distinguished by the larger body size, wider pronotum, and fewer femoral and tibial spines than those of its congener. Concerning the males, the two black lateral borders of the wide white band in the middle of discoidal area are pointing in opposite directions in *T. xishiae* sp. nov. (Fig. 13D), however, are parallel in *T. sinecus* (Fig. 13B). The structure of the female’s abdomen is also different from that of its congeners (Fig. 9A): lateral margins of 4^th^-7^th^ abdominal tergites each bear a distinctly expanded lobe; abdomen yellowish white, lateral margins of 3^th^-5^th^ abdomen tergite with green plaques; 6^th^-7^th^ completely white.

This beautiful species is distributed in the southern Himalayas, which is the northernmost and westernmost record for *Theopropus*.

###### Distribution.

China: Tibet, Medog. Expected to also occur in N India.

###### Etymology.

The new species was named after Xi Shi who was born in The Spring-Autumn Period, the top of the four beautiful women in ancient China, the beauty representative in Chinese culture.

##### Theopropus

Taxon classificationAnimaliaMantodeaHymenopodidae

sp.

ABC83524-5073-58D8-941B-968DE849473C

Figs 2C ; 3C ; 5C, F ; 6C ; 7C ; 8C, F ; 9C, E ; 10C ; 12 ; 13E, F ; 14F

###### Material examined.

35♂, 6♀. **China** • 5♂; Yunnan, Jinghong, Xiaopuxi; 22°01'52"N, 100°58'19"E; 1100 m; 10-V-2019; Chao Wu leg.; CWC; • 7♂; Yunnan, Jinghong, Menglun; 21°57'37"N, 101°12'17"E; 850 m; 6-V-2019; Chao Wu leg.; IZCAS; • 8♂; Yunnan, Mengla, Bubeng; 21°37'02"N, 101°34'44"E; 900 m; 11-X-2014; Chao Wu leg.; CWC; • 15♂; Yunnan, Mengla, Mohan; 21°11'04"N, 101°43'31"E; 1000 m; 30-IX-2017; Chao Wu leg.; CWC; • 1♀; Yunnan, Jinghong, Menglun; 21°57'37"N, 101°12'17"E; 850 m; 5-X-2014; Chao Wu leg.; IZCAS; • 1♀; Yunnan, Mengla, Mohan; 21°11'04"N, 101°43'31"E; 1000 m; 22-IX-2017; Chao Wu leg.; CWC; • 1♀; Yunnan, Jinghong, Menglun; 21°57'37"N, 101°12'17"E; 850 m; 22-IX-2013; Chao Wu leg.; CWC; • 1♀; Yunnan, Jinghong, Damenglong; 21°30'43"N, 100°40'22"E; 600 m; 10-X-2013; Chao Wu leg.; CWC.

**Thailand** • 2♀; Thailand; Chiang Mai; VII-2017; Nan Jiang leg.; CWC.

###### Comments.

**Male**. Compound eyes oval, anteriorly protruding. Prolongation bifid vertex conical, not reaching imaginary line extending between the apexes of the eyes (Fig. 5C). Lateral pronotal expansion wide, ratio of pronotum length to supracoxal dilatation width about 1.51–1.53. Lateral margins of pronotum bearing inconspicuous teeth. Black band on each lateral margin of metazone continuous (Fig. 6C). Anterior coxa bearing 8–10 dorsal spines, femora with 17 anteroventral spines, tibia with 17–18 anteroventral and 21 posteroventral spines; tibia with two black horizontal bands on ventral side (Fig. 8C, F). Forewings green; the black lateral borders of the white band in middle of discoidal area wide, blurry. Hindwings orangish red, transparent.

***External genitalia*:** Simple. Left phallomere wide, rhomboidal; secondary distal process (spd) indistinct; phalloid apophysis (afa) short; posterior process of left phallomere (paa) digitiform (Fig. 10C).

**Female.** Large-sized, robust. Ratio of pronotum length to supracoxal dilatation width about 1.37–1.40; black band on lateral margin of metazone continuous (Fig. 7C). Lateral margins of pronotum bearing prominently serrated teeth. Hindwings yellow, transparent at edges.

###### Differential diagnosis.

Compared with the other two species of *Theopropus* in China, this species is smaller in body size, the prolongation on the vertex is small in the female, and the difference in body size between the sexes is more pronounced. The dorsal spines on anterior coxae are larger and longer than those of the other two species. The anterior tibia does not have a black spot near the spur. The male characteristics are also close to that of *T. cattulus* (Westwood, 1889) (type locality in Java, Indonesia) but the markings of the pronotum and forewings are different. In addition, in this species, the male hindwings do not have the opaque area which is present in the male specimens of *T. elegans* from the Malay Peninsula. These specimens may represent another new species, and further research on this species is needed.

###### Measurements

**(length in mm).** Body (head to wings): male 24.8–25.3, female 41.2–42.0; body (vertex to abdomen end): male 20.4–21.8, female 38.5.1–49.8; pronotum: male 5.1–5.2, female 10.8–10.9; fore coxae: male 5.0–5.1, female 12.6–13.0; fore femora: male 5.7–5.8, female 14.1–14.3; fore tibiae: male 4.8–4.9, female 11.0–11.2; middle femora: male 4.9–5.0, female 10.7–10.9; hind femora: male 5.8–5.9, female 11.6–11.8; forewing: male 17.1–17.4, female 27.2–27.6; hindwing: male 15.6–16.1, female 23.6–23.8.

###### Distribution.

China: Yunnan; Thailand.

###### Biological characteristics.

*Theopropus* species often live among flowers. In Huaping of Guangxi Province (southwestern of China), *T. sinecus sinecus* often appears among the inflorescences of Valerianaceae plants, the mottling pattern of the mantis allows them to blend in such an environment (Fig. 13A, B). The males have phototaxis during night time.

In China, the genus *Theopropus* ranges northwestwards to Medog, Tibet, and eastwards to the central Fujian Province. *Theopropus* species often inhabit medium-elevation forests. In Guangxi Province, *T. sinecus sinecus* is distributed at an altitude of about 800–1400 m. *Theopropus sinecus qiongae* ssp. nov. was collected from 800–1000 m in Hainan Island. *Theopropus* species in Yunnan Province were collected from 600–1100 m. *Theopropus xishiae* sp. nov. from Tibet were collected from 900–1400 m. *Theopropus sinecus sinecus* overwinters as eggs or nymphs in Guangxi, Fujian, and Guangdong Provinces. In the mountains of these areas, it snows in winter, and the lowest temperature about -5~-10 °C. Nymphs of *T. sinecus sinecus* hide in the deciduous layers during winter, and begin to grow about April of the following year; adults can be seen from July to November. In Hainan Island, no clear seasonality patterns can be observed in *T. sinecus qiongae* ssp. nov., for which adults and nymphs can be found in each season. The same situation is found in southern Yunnan. The situation for *T. xishiae* sp. nov. in Medog of Tibet is unclear, but adults of *T. xishiae* can be seen from July to October.

In rare instances, females of *Theopropus sinecus* collected from Guangdong and Hainan have been discovered to be parasitized by horsehair worms.

## Discussion

Hainan Island is an isolated island in southeastern China. The Qiongzhou Strait between Hainan Island and the mainland is a geographical barrier for the separation and differentiation among closely-related species which separately live on either side of the barrier, as is the case postulated for *T. s. sinecus* and *T. s. qiongae* ssp. nov.. The ancestor of *T. xishiae* sp. nov. in the Himalayas is speculated to come from the Assam Region, and numerous mountains have become obstacles which prevented them from spreading northwards. However, in southern Yunnan, the distribution boundaries of *T. s. sinecus* and *T.* sp. are not yet clear, the two species seem to be separated in the Honghe area; *T. s. sinecus* lives in the eastern part, and *T.* sp. lives in the western part.

The structure of the male genitalia of *Theopropus* is rather simple compared to other species within the order Mantodea. Nonetheless, the shape of the ventral phallomere can be used to distinguish the three species in China: the ventral phallomere is wide and secondary distal process (spd) indistinct in *T. sinecus*; it is wide and with inconspicuous secondary distal process in *T. xishiae* sp. nov.; and it is rhomboidal in *T.* sp.

## Conclusion

After examining numerous specimens, which were collected in China and neighboring countries, we reached the following conclusion. *Theopropus sinecus* Yang is valid. Those specimens that were collected from Hainan Island and identified as *T. cattulus* Westwood by Zhu et al. (2012) belong to a new subspecies for *T. sinecus*, i.e., *T. sinecus qiongae* ssp. nov.. The male specimen, which was identified as *T. elegans* in Wuyishan, Fujian Province by Wang (1993) should also be identified as *T. sinecus*. A unique new species, *T. xishiae* sp. nov. was discovered in the Himalayas (Tibet in China). In addition, numerous specimens of *Theopropus* from southern Yunnan are temporarily assigned to an unidentified species not identical with the previous two, and also not identical with *T. elegans*. The species *T. elegans* is not distributed in China.

## Supplementary Material

XML Treatment for Theopropus

XML Treatment for Theopropus sinecus

XML Treatment for Theopropus sinecus sinecus

XML Treatment for Theopropus sinecus qiongae

XML Treatment for Theopropus xishiae

XML Treatment for Theopropus
